# Diagnostic relevance of Humanin, GAS5 and miR-21/miR-103 in prostate disease risk stratification

**DOI:** 10.1007/s10238-025-01810-z

**Published:** 2025-08-06

**Authors:** Donatella Coradduzza, Sara Cruciani, Leonardo Sibono, Alessandro Tedde, Angelo Zinellu, Margherita Maioli, Alessio Aligi Cogoni, Maria Rosaria De Miglio, Serenella Medici, Massimo Madonia, Andrea Angius, Massimiliano Grosso, Ciriaco Carru

**Affiliations:** 1 Department of Biomedical Sciences, University of Sassari, Viale San Pietro 43/B, 07100 Sassari, Italy; 2 Department of Mechanical, Chemical, and Materials Engineering, University of Cagliari, Cagliari, Italy; 3 Department of Medicine, Surgery and Pharmacy, University of Sassari, Sassari, Italy; 4 Unit of Urology, University Hospital of Sassari (A.O.U. SS), Sassari, Italy; 5 Medical Oncology Unit, University Hospital (AOU) of Sassari, 07100 Sassari, Italy; 6 Department of Chemical, Physical, Mathematical and Natural Sciences, University of Sassari, Sassari, Italy; 7 Institute of Genetic and Biomedical Research (IRGB), National Research Council (CNR), Cittadella Universitaria Cagliari, 09042 Monserrato, CA Italy

**Keywords:** Biomarkers, Clinical laboratory, Exosomal miRNAs, Humanin, Non-invasive diagnosis, Prostate cancer

## Abstract

This study aimed to evaluate the diagnostic significance of circulating mitochondrial-derived peptides, Humanin and MOTS-c, the long non-coding RNA GAS5, and exosomal microRNAs miR-21 and miR-103 in stratifying prostate diseases, including benign prostatic hyperplasia (BPH), precancerous lesions (PL), and prostate cancer (PCa). These biomarkers were selected based on their established roles in cellular stress responses, apoptosis regulation, inflammation, and tumor progression. A cohort of 375 male patients suspected of prostate cancer were enrolled. Plasma and exosomal levels of Humanin, MOTS-c, GAS5, miR-21, and miR-103 were measured. Diagnostic performance was assessed using receiver operating characteristic (ROC) curve analysis, principal component analysis (PCA), partial least squares discriminant analysis (PLS-DA), and decision tree models. Results showed significant downregulation of Humanin and GAS5 in both PL and PCa compared to BPH, supporting their role in early disease transition. Exosomal miR-21 and miR-103 were significantly upregulated in PCa, with miR-21 exhibiting outstanding discriminative power between BPH and PL (AUC = 1.000) and between PL and PCa (AUC = 0.9932). MOTS-c, a mitochondrial-derived peptide, displayed elevated levels in PL compared to BPH, suggesting its involvement in early malignant transformation. A plasma-only diagnostic model combining Humanin, GAS5, and MOTS-c reached 95% cross-validated classification accuracy across clinical groups. Combination of circulating Humanin, MOTS-c, GAS5, and exosomal miRNAs provides a promising non-invasive biomarker panel for risk stratification in prostate diseases. This integrated molecular approach may enhance diagnostic precision and guide personalized clinical decision-making in prostate cancer management.

**Graphical abstract:**

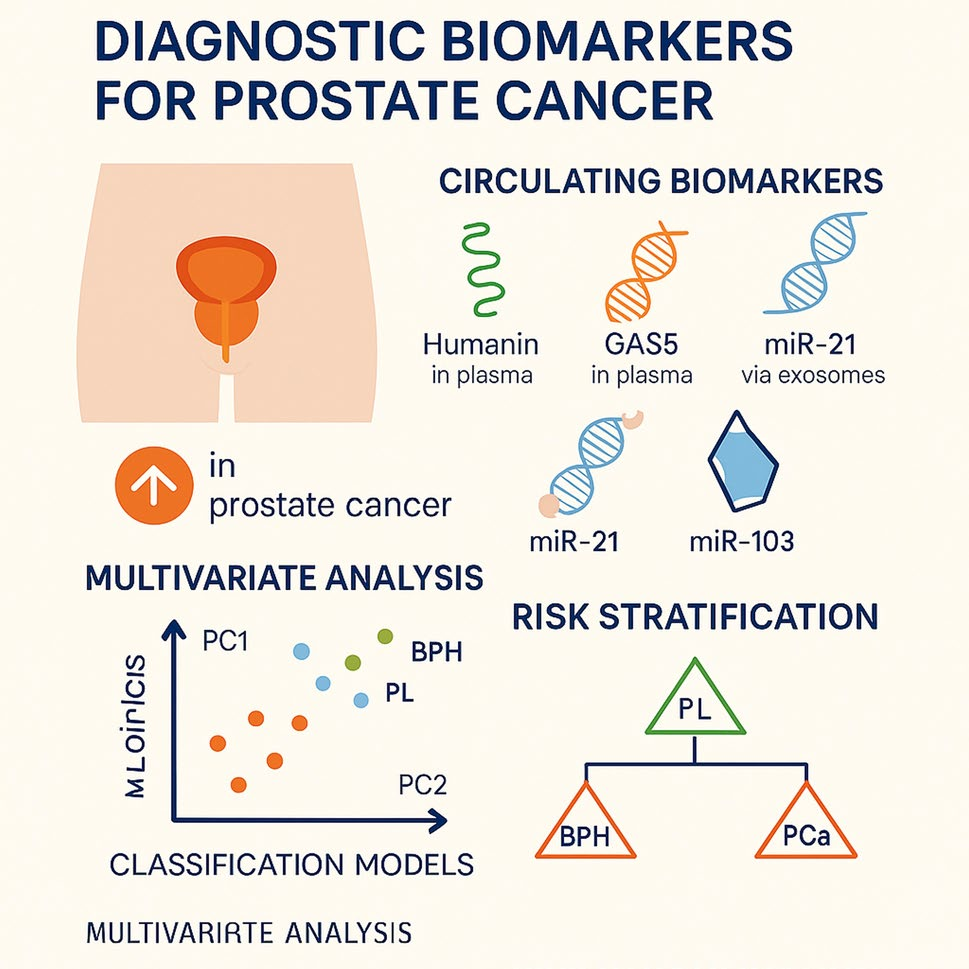

**Supplementary Information:**

The online version contains supplementary material available at 10.1007/s10238-025-01810-z.

## Introduction

Prostate cancer (PCa) is the second most common cancers in men, accounting for 15% of all cancers worldwide [1]. Its high incidence and mortality make it a major public health concern [2, 3]. Projections from the Lancet Commission estimate that new cases will nearly triple by 2040, reaching almost three million [4, 5]. PCa is a multifactorial disease driven by complex network between biochemical regulation [6], genetic predispositions and environmental factors like diet and physical activity [7–9]. Traditional diagnostics tools like digital rectal examination (DRE) [10] and prostate-specific antigen (PSA) testing remain essential but are limited by low specificity and sensitivity [11, 12], leading to overdiagnosis and underdiagnosis [2, 13]. Emerging technologies, such as multiparametric magnetic resonance imaging (mpMRI), offer greater promise but still need further validation [14]. Treatment strategies range from active surveillance to radical prostatectomy and radiation therapy, increasingly guided by personalized assessments of tumor and patient characteristics [15]. This has driven research toward identifying new biomarkers [16–18], with growing interest in mitochondrial peptides and their role in cancer-related metabolic pathways[19]. Mitochondrial dysfunction is increasingly recognized as a hallmark of cancer [20], contributing to altered metabolism, resistance to apoptosis, and oxidative stress [20, 21]. Cancer cells characteristics, such as resistance to apoptosis, excessive production of reactive oxygen species (ROS) [22], and altered oxidative phosphorylation, are closely related to mitochondrial functions [23, 24]. In this context, mitochondrial-derived peptides (MDPs) like Humanin and MOTS-c, have gained attention for their cytoprotective roles [25, 26]. Humanin, a small peptide with broad tissue expression, modulates oxidative stress, inflammation, and apoptosis, potentially influencing tumor behavior [27]. Humanin is a 24 amino acid peptide discovered in 2001, known for its neuroprotective and cytoprotective properties [28, 29]. It interacts with various cellular pathways to mitigate oxidative stress, apoptosis, and inflammation [30]. Humanin is expressed in several tissues (heart, kidneys, liver, testes, skeletal muscles, and brain) and can be found in plasma, cerebrospinal and seminal fluid [31]. It represents a ligand for membrane receptors, activating cytoprotective signaling pathways and promoting autophagy [32]. Understanding the interaction between prostate cancer and humanin is mandatory: its ability to modulate different metabolic pathways suggests it could influence the development and progression of PCa [19, 33]. Humanin interacts with regulatory molecules, such as microRNAs (miRNAs) miR-21 and miR-103 [34]. These miRNAs are known for their role in cancer cell proliferation and survival, and their modulation by humanin could impact prostate cancer outcomes [35]. MOTS-c, a MDP encoded by the mitochondrial 12S rRNA, acts as a metabolic regulator of glucose and lipid homeostasis and has been shown to exert anti-proliferative effects in cancer models [36]. Despite limited studies in PCa, its role in modulating key signaling pathways related to cell growth and stress adaptation suggests a potential diagnostic value in early disease transitions such as precancerous lesions [37, 38]. Parallel to this, non-coding RNAs (lncRNAs) are emerging as key regulatory molecules in cancer [39, 40]. The long non-coding RNA GAS5(Growth Arrest-Specific 5), is known for its tumor-suppressive function, often downregulated in PCa [41–43]. GAS5 is crucial in regulating cell growth and apoptosis [44, 45]. Its expression is often reduced in tumor tissues, suggesting a suppressive function against cancer [46]. GAS5 acts as a molecular sponge for oncogenic microRNAs such as miR-21 and miR-103—both implicated in cancer progression [44, 45, 47, 48]. MiR-21, a well-characterized oncomiR, promotes tumor proliferation and resistance to apoptosis in various cancers, including PCa. Similarly, miR-103 has been implicated in epithelial-to-mesenchymal transition (EMT), invasion, and chemoresistance [45]. The interplay between Humanin, MOTS-c, GAS5, and these miRNAs is of particular interest: they may operate within a regulatory axis affecting mitochondrial metabolism, inflammation, and tumor progression. Recent findings suggest that long non-coding RNA GAS5 and the microRNAs miR-21 and miR-103 contribute to the post-transcriptional regulation of cytoprotective mitochondrial peptides [49, 50], such as Humanin and MOTS-c [51, 52]. These non-coding RNAs appear to modulate the expression or stability of mitochondrial-derived peptides through complex molecular interactions [53]. For instance, GAS5 may function as a competitive endogenous RNA, sequestering miR-21 and miR-103 and thereby influencing the translational efficiency or degradation of transcripts encoding mitochondrial peptides [49, 54–57]. This regulatory network introduces an additional layer of control over mitochondrial function, potentially affecting cellular pathways involved in growth arrest, apoptosis, and stress resistance—key processes in prostate carcinogenesis. The selection of these molecular markers is therefore grounded in their shared relevance to mitochondrial function, apoptosis regulation, and non-coding RNA-mediated oncogenic signaling—three fundamental mechanisms driving prostate cancer development. This study explores their potential as a panel of circulating biomarker capable of distinguishing between benign prostatic hyperplasia, precancerous lesions, and prostate cancer, underscoring their translational value in precision diagnostics.

## Materials and methods

### Study design

We studied a cohort of 375 male patients with clinical suspicion of prostate cancer (PCa), enrolled at the Urology Clinic of the University Hospital of Sassari. All participants underwent a standardized diagnostic workflow consistent with the guidelines of the International Society of Urological Pathology (ISUP). We distinguish between benign prostatic hyperplasia (BPH), precancerous lesions (PL), and confirmed PCa cases based on clinical, imaging, and histological criteria. Eligibility criteria included men over the age of 50 with clinical or biochemical indications of prostate pathology—such as elevated prostate-specific antigen (PSA) levels, abnormal digital rectal examination (DRE), or radiological suspicion of malignancy [58, 59]. Patients with a prior diagnosis of PCa, those undergoing or having completed treatment for any malignancy, individuals with severe comorbidities contraindicating biopsy, or those with incomplete clinical records were excluded. Peripheral blood samples were collected in EDTA-coated tubes prior to digital rectal examination (DRE), to prevent procedure-related alterations in plasma or exosomal biomarker levels. Biological samples were collected for each subject before biopsy procedures as part of the diagnostic algorithm. The study was approved by the Independent Ethics Committee of the Azienda Ospedaliero Universitaria di Cagliari (Approval Code: Prot. PG/2022/4985; Approval Date: 30/03/2022), in accordance with the Declaration of Helsinki and international standards for good clinical practice. Written informed consent was obtained from all participants.

The diagnostic workflow included:

- Comprehensive clinical evaluation, including DRE.
- Serum PSA measurement.
- Multiparametric magnetic resonance imaging (mpMRI), interpreted according to PI-RADS v2.0 criteria.
- Transrectal ultrasound (TRUS)-guided prostate biopsy, performed in 100% of patients, with a minimum of 12 cores per case.
- Histopathological analysis of biopsy samples for definitive diagnosis.
- Biopsy samples were assessed using Gleason grading and classified according to the ISUP Grade Groups. The final diagnosis—either BPH, PL, or PCa—was based on histological confirmation and served as the endpoint of this study. Gleason score, prostate volume, and PI-RADS were recorded for diagnostic purposes but were not included in the present analysis, which focused on the diagnostic stratification of BPH, PL, and PCa based on circulating biomarkers.

### Exosomes isolation

Total Exosome Isolation Reagent Kit (Thermo Fisher Scientific, Grand Island, NY, USA) was used for exosomes isolation from plasma of BPH, PL and PCa patients according to the manufacturer’s instructions.

### miRNA expression

RNA extraction from plasma and from exosomes was performed using miRNeasy Serum/Plasma Kit (Qiagen, Germany, Cat. No. 217184) and miRNeasy Mini Kit (Qiagen, Germany, Cat. No. 217004), respectively. miRNA expression was evaluated using the TaqMan® MicroRNA Reverse Transcription Kit (Thermo Fisher Scientific, Grand Island, NY, USA, Cat. No. 4366596), followed by quantitative PCR using TaqMan® MicroRNA Assays specific for hsa-miR-21-5p (Assay ID: 000397) and hsa-miR-103a-3p (Assay ID: 000439) (Applied Biosystems, Thermo Fisher Scientific). Ct values were normalized to RNU6-1 (Assay ID: 001973), described in Table 1.

**Table 1:** MiRNA accession numbers, symbols, and sequences of analyzed RNAs and peptides

Accession ID number	Symbol
MI0000077	hsa-miR 21-5p
MI0000109	hsa-miR-103a-3p
U6 snRNA	RNU6-1
ENSG00000234741	GAS5
ENST00000229239.10	GAPDH (hGAPDH)
ENSG00000238796	MTRNR2L1 (Humanin)
ENSG00000273398	MTRNR1 (MOTS-c)

### Gene expression analysis

Total RNA of each sample was extracted from plasma and exosomes using RNeasy Mini Kits for RNA Purification (Qiagen, Germany) and then quantified by the NanoDrop™ One/OneC Microvolume UV–Vis spectrophotometer (Thermo Fisher Scientific, Grand Island, NY, USA). RNA samples were reverse-transcribed using High-Capacity cDNA Reverse Transcription Kit (Applied Biosystems, Thermo Fisher Scientific, USA). qPCR was performed using SYBR™ Green PCR Master Mix (Applied Biosystems, Thermo Fisher Scientific, USA), in a CFX Thermal Cycler (Bio-Rad, Hercules, CA, USA). LncRNA-GAS5 Ct values were normalized to hGAPDH, as a reference gene. The primers used (Thermo Fisher Scientific, Grand Island, NY, USA), are described in Table 2.

**Table 2:** Primer sequences

Primer name	Forward	Reverse
hGAPDH	GAGTCAACGGAATTTGGTCGT	GACAAGCTTCCCGTTCTCAG
GAS5	CTTGCCTGGACCAGCTTAAT	CAAGCCGACTCTCCATACCT

### ELISA

Concentrations of Humanin, and Mitochondria-derived peptide (MOTS-c) were evaluated by ELISA using Human Humanin ELISA Kit (Assay Genie, Dublin, Ireland) and MOTS Enzyme-linked Immunosorbent Assay Kit (Cloud-Clone Corp., Usa) respectively, following the manufacturer’s instructions. 100 uM of standards and samples were added to each well. Each sample was assayed in duplicate, and values were expressed as the mean ±SD of 2 measures per sample.

### Statistical analysis

Data analysis was conducted using both univariate and multivariate statistical approaches to ensure the robust identification of relevant biomarkers. Analyses were performed using GraphPad Prism 10.0 (GraphPad Software, San Diego, CA, USA), R software (v.4.2.3), and Python (Scikit-learn v.3.10). Continuous variables were expressed as means ± standard deviation (SD) or medians with interquartile ranges (IQR), based on distribution normality assessed via the Shapiro–Wilk test. Categorical variables were reported as frequencies and percentages. Comparisons among the three diagnostic groups (BPH, PL, PCa) were conducted using the Kruskal–Wallis test, one-way ANOVA with Tukey’s post hoc correction, or Wilcoxon signed-rank test, where appropriate. A two-tailed p-value < 0.05 was considered statistically significant. The diagnostic performance of the molecular biomarkers was assessed using Receiver Operating Characteristic (ROC) curve analysis. To assess whether differences between the AUC values of individual biomarkers were statistically significant, DeLong’s test was employed [60]. This non-parametric method allows for the comparison of ROC curves derived from the same sample set. A significance level of 5% was selected as the threshold for determining statistical significance.

To analyze distribution patterns and identify potential group-level differences in biomarker profiles, we conducted PCA [61]. Multivariate analyses were performed under two conditions: one incorporating exosomal markers and one excluding them. The reason for omitting exosomal markers in the latter analysis is explained in detail in the Results section. In both cases data where subjected to zero mean and unity variance standardization prior to performing PCA.

To further evaluate the discriminative capacity of the biomarker panel, we then applied Partial Least Squares Discriminant Analysis (PLS-DA), a supervised multivariate technique widely used in omics and biomedical research [62]. PLS-DA combines features of principal component analysis with class prediction, allowing for optimal separation of predefined groups while handling multicollinearity among variables. The PLS-DA model performance was assessed via Monte Carlo cross-validation (1000iterations). VIP scores were also computed with the aim to measure the importance of each variable in the PLS-DA model. Higher VIP scores indicate that the variable has a greater influence on the classification. Biomarkers with Variable Importance in Projection (VIP) scores exceeding 1.0 were considered to have substantial discriminative relevance [63]. Finally, a Classification and Regression Tree (CART) model is implemented. The CART algorithm is a univariate decision tree learning method that recursively partitions the feature space by evaluating one variable at a time [64]. One of the main advantages of tree-based models is their interpretability; they can be visualized as graphical structures, making the decision-making process intuitive and easy to follow. This method is widely used in clinical diagnostics [65, 66]. Here, a CART classifier was developed using the most informative plasma biomarkers, achieving a classification accuracy of 95% based on three variables: Humanin, MOTS-c, and GAS5. The Gini index is used as the default splitting criterion. Multivariate statistical analyses and CART modeling were supported within MATLAB R2024b environment using both custom scripts developed by the authors and Classification Toolbox v.7.0 [67].

## Results

### Clinical and laboratory parameters

A total of 375 patients were included and stratified into three diagnostic groups based on prostate biopsy: BPH (n = 150), PL (n = 75), and PCa (n = 150). The overall mean age of participants was 69.34 ± 7.89 years. The mean age in the BPH group was 67.73 ± 7.95 years, in the PL group was 66.95 ± 7.88 years, and in the PCa group was 72.18 ± 7.08 years. A detailed overview of clinical, demographic, and laboratory characteristics is reported in Table 3. The primary objective of this study was to evaluate the relationship between circulating Humanin levels, Long Non-Coding RNA GAS5, microRNA-21 (miR-21), microRNA-103 (miR-103), and prostate cancer risk. Patients diagnosed with PCa exhibited higher PSA values and lower Index % compared to BPH and PL groups. Additionally, significant differences were observed across groups for hemoglobin (HGB), red cell distribution width (RDW), hemoglobin distribution width (HDW), and inflammatory indices such as neutrophil-to-lymphocyte ratio (NLR) and International Index of Erectile Function (IIEF), supporting a more advanced disease profile in PCa patients.

**Table 3:** Clinical, Demographic, and Laboratory Variables in Patients with BPH, PL, and PC. * p < 0.05 is considered statistically significant

Variable	BPH (n = 150)	PL (n = 75)	PCa (n = 150)	*p* value
AGE	67.00 (63.00–73.00)	68.00 (60.00–74.00)	74.00 (68.00–77.00)	1.2e-07*
PSA	5.31 (3.44–8.67)	5.24 (3.13–6.83)	7.61 (5.34–13.94)	3.1e-08*
INDEX_%	18.00 (13.00–22.00)	16.00 (7.00–23.00)	13.00 (9.00–20.25)	0.0516
WBC	6.95 (5.84–7.92)	6.91 (5.80–8.21)	6.84 (5.90–8.44)	0.7324
RBC	5.12 (4.81–5.59)	5.16 (4.87–5.53)	5.01 (4.58–5.47)	0.0526
HGB	14.80 (13.72–15.50)	14.90 (14.15–15.75)	14.00 (12.90–14.90)	1.2e-06*
RDW	13.60 (13.00–14.70)	13.50 (13.10–14.35)	13.90 (13.10–15.35)	0.0238*
HDW	2.60 (2.40–2.80)	2.50 (2.30–2.70)	2.60 (2.40–2.90)	0.0125*
MPV	8.65 (8.00–9.10)	8.60 (8.05–9.30)	8.70 (8.00–9.28)	0.8234
PLT	225.97 ± 53.53	221.01 ± 48.78	223.29 ± 62.44	0.8120
PCT	1.90 (1.60–2.24)	1.85 (1.56–2.18)	1.92 (1.58–2.24)	0.7826
NEUTROPHILES	3.90 (3.23–4.80)	3.90 (3.20–5.20)	4.20 (3.40–5.10)	0.2818
LYMPHOCYTES	2.00 (1.50–2.50)	1.90 (1.60–2.42)	1.90 (1.50–2.40)	0.4430
MONOCYTES	0.50 (0.40–0.50)	0.50 (0.40–0.60)	0.50 (0.40–0.50)	0.6125
EOS#	0.20 (0.10–0.20)	0.20 (0.10–0.20)	0.20 (0.10–0.20)	0.6682
BASO#	0.00 (0.00–0.10)	0.00 (0.00–0.10)	0.00 (0.00–0.10)	0.3019
LUC#	0.10 (0.10–0.20)	0.10 (0.10–0.20)	0.10 (0.10–0.20)	0.4980
LUC%	1.90 (1.60–2.40)	1.80 (1.50–2.50)	1.80 (1.50–2.40)	0.6870
LMR	0.26 (0.19–0.43)	0.32 (0.21–3.14)	0.27 (0.20–0.41)	0.0996
NLR	1.96 (1.50–2.70)	1.93 (1.53–2.41)	2.27 (1.78–3.08)	0.0469*
PLR	112.90 (86.28–146.80)	113.68 (82.33–150.00)	111.71 (86.87–148.22)	0.7855
IIEF	20.00 (14.00–22.00)	19.00 (12.75–22.00)	16.00 (9.00–20.00)	0.0042*

Clinical, demographic, and laboratory variables in patients diagnosed with benign prostatic hyperplasia (BPH), precancerous lesions (PL), and prostate cancer (PCa). Continuous variables are reported as median and interquartile range (IQR) or mean ± standard deviation (SD), as appropriate. Statistical comparisons were performed using Kruskal–Wallis or ANOVA with *p* < 0.05 considered statistically significant.

AGE, age in years; PSA, prostate-specific antigen (ng/mL); INDEX_%, percentage of free PSA relative to total PSA (f/t ratio), expressed as a percentage; WBC, white blood cell count (× 10^9^/L); RBC, red blood cell count (× 10^12^ /L); HGB, Hemoglobin concentration (g/dL); RDW, red cell distribution width, indicating variability in red cell size (%); HDW, hemoglobin distribution width, reflecting variability in hemoglobin concentration among red cells (%); MPV, mean platelet volume, a marker of platelet activation (femtoliters); PLT, platelet count (× 10^9^/L); PCT, plateletcrit, representing the volume percentage of platelets in blood (%); NEUTROPHILES, absolute neutrophil count (× 10^9^/L); LYMPHOCYTES, absolute lymphocyte count (× 10^9^/L); MONOCYTES, absolute monocyte count (× 10^9^/L); EOS#, absolute eosinophil count (× 10^9^/L); BASO#, absolute basophil count (× 10^9^/L); LUC#, absolute count of large unstained cells (× 10^9^/L), typically representing activated lymphocytes or atypical cells; LUC%, percentage of large unstained cells out of total leukocytes; LMR, lymphocyte-to-monocyte ratio, an inflammatory marker; NLR, neutrophil-to-lymphocyte ratio, used as a prognostic indicator in cancer; PLR, platelet-to-lymphocyte ratio, an index of systemic inflammation; IIEF, international index of erectile function, a validated questionnaire assessing erectile function*.*

#### MiRNA expression

**Fig. 1:**
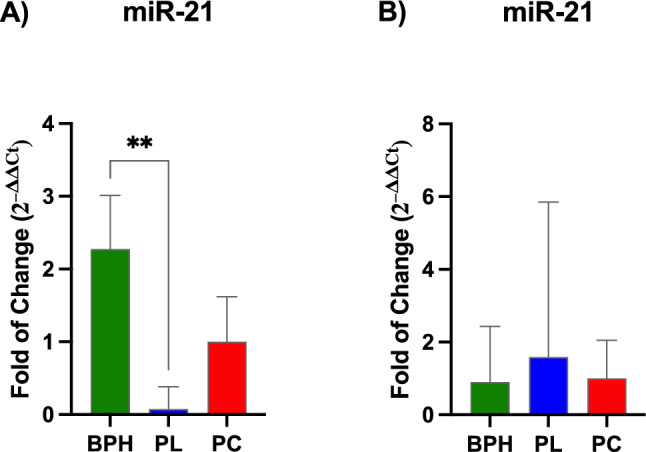
Expression of miR-21 in plasma and exosomes. The expression of miR-21 in plasma (Panel A) and exosomes (Panel B) were evaluated BPH, PL, and PCa patients. The mRNA levels for each gene were normalized to U6snRNA. Data are expressed as mean ± SD (**p ≤ 0.01)

**Fig. 2:**
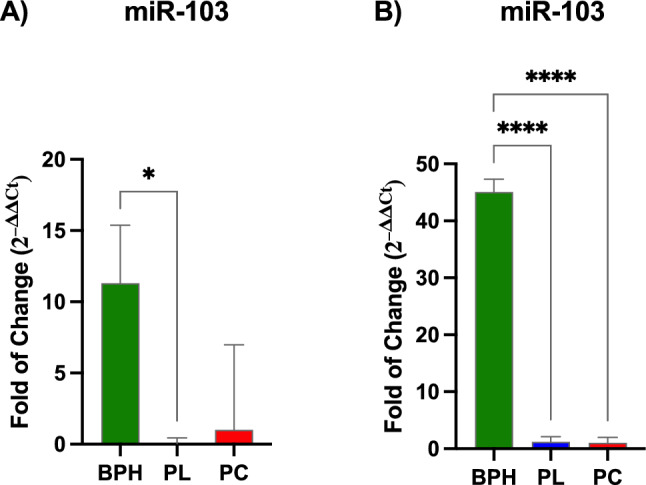
Expression of miR-103 in plasma and exosomes. The expression of miR-103 in plasma (Panel A) and exosomes (Panel B) was evaluated BPH, PL, and PCa patients. The mRNA levels for each gene were normalized to U6snRNA. Data are expressed as mean ± SD (*p ≤ 0.05), (****p ≤ 0.0001)

MiR-21 and miR-103 expression were evaluated in plasma and exosomes of BPH, PL and PCa patients. As shown in Fig. 1, miR-21 was significantly upregulated in plasma (Panel A) of BPH patients, as compared to PL and PCa, while no significative difference was evidenced in exosomes. miR-103 (Fig. 2) showed a similar trend, whose levels were significantly upregulated in plasma of BPH patients (Panel A), as compared to PL, and in exosomes (Panel B), as compared to both PL and PCa patients.

#### LcnRNA expression

**Fig. 3:**
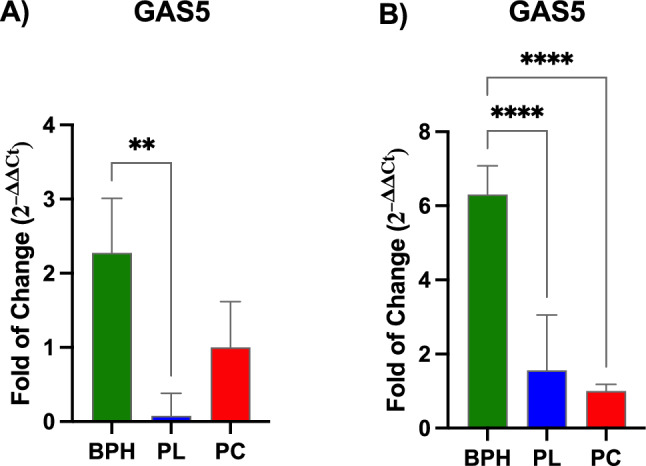
Expression of Lnc-RNA-GAS5. The expression of GAS5 was evaluated in plasma and exosomes of BPH, PL and PCa patients by qPCR. The levels of expression of GAS5 were normalized to Glyceraldehyde-3-Phosphate-Dehydrogenase (GAPDH). Data are expressed as mean ± SD (**p ≤ 0.01), (****p ≤ 0.0001)

Figure 3 shows the levels of expression of Lnc-RNA-GAS5 in plasma and exosomes of BPH, PL and PCa patients. The expression of GAS5 was significantly increased in both plasma (Panel A) and exosomes (Panel B) of BPH patients, as compared to PL patients. In addition, GAS5 was significantly increased expressed in exosomes of BPH patients, as also compared to PCa patients.

#### ELISA assay

**Fig. 4:**
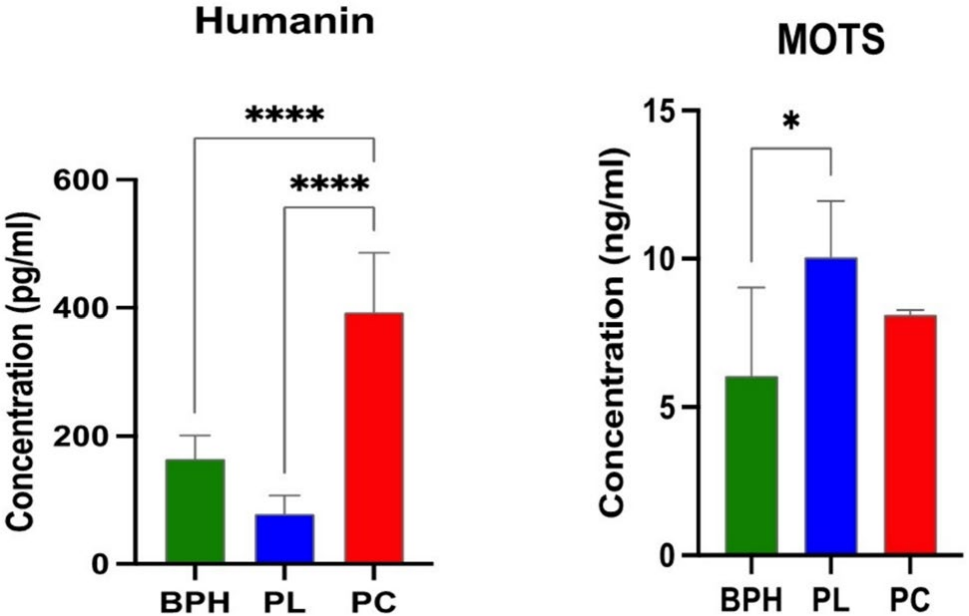
Humanin, and MOTS secreted levels in plasma samples were determined by ELISA. Data are expressed as mean ± SD referred to the control (*p ≤ 0.05), (***p ≤ 0.001), (****p ≤ 0.0001)

Humanin ELISA (Fig. 4) revealed significantly increased levels in the plasma of PCa patients, compared with PL and BPH samples. No significant differences were shown between PL and BPH patients. The MOTS-c ELISA (Fig. 4) revealed significantly increased levels of the peptide in patients with PL, compared with those with BPH, while no significant differences were observed between PL and PCa and between patients with BPH and PCa.

### Diagnostic performance of individual biomarkers (AUC analysis)

To evaluate the diagnostic utility of the investigated biomarkers, ROC curve analyses were performed comprising all pairwise among the clinical groups: BPH, PL, and PCa. Table 4 summarizes the area under the curve (AUC) values for Humanin, MOTS, miR-21 (plasma and exosome), miR-103 (plasma and exosome), and GAS5 (plasma and exosome). The related ROC curves are reported in the Supplementary Material, Section S1. The comparison between BPH and PL revealed outstanding discriminatory ability (AUC = 1.000) for exo-miR-21 and exo-miR-103 (AUC = 0.9973), with similarly high values for Humanin (0.9855), plasma-miR-10 (0.9817), and plasma-GAS5 (0.9556). These results suggest that these molecules can effectively differentiate benign from potentially premalignant conditions. In the PL versus PCa comparison, Humanin maintained high diagnostic performance (AUC = 0.9867), alongside exo-miR-21 (0.9932), plasma-miR-103 (0.9763), and plasma-GAS5 (0.8711), confirming their potential to distinguish early from established malignancy. For BPH versus PCa, exo-miR-103 again exhibited excellent discriminatory capacity (AUC = 0.9986), followed by MOTS (0.9115) and exo-miR-21 (0.8593). However, Humanin (0.5835) and plasma-miR-103 (0.5747) displayed limited effectiveness in this context.

**Table 4:** AUC analysis from individual variables

GROUP	Humanin	MOTS	miR-21 Plasma	miR-21 Exosome	miR-103 Plasma	miR-103 Exosome	GAS5 Plasma	GAS5 Exosome
BPH versus PL	0.9855	0.8876	0.7234	1	0.9817	0.9973	0.9556	0.6588
PL versus PCa	0.9867	0.7675	0.5325	0.9932	0.9763	0.5404	0.8711	0.5256
BPH versus PCa	0.5835	0.9115	0.7474	0.8593	0.5747	0.9986	0.7611	0.6432

Table 5 shows the results of the AUC analysis when combining two clinical groups. Noteworthy, miR-103 Exosome is the only marker that provides satisfactorily performances in discriminating BPH+ PL from PCa. On the other hand, MOTS, miR-21 Exosome and miR-103 Exosome provided high sensitivity, with the latter variable showing the greatest value. Detailed results of Delong’s test are reported in the Supplementary Material, Section S2.

**Table 5:** Receiver operating characteristic (ROC) AUC analysis for composite group comparisons: BPH + PL versus PCa, and BPH versus PL + PCa, to evaluate marker performance across differential diagnostic scenarios

GROUP	Humanin	MOTS	miR-21 Plasma	miR-21 Exosome	miR-103 Plasma	miR-103 Exosome	GAS5 Plasma	GAS5 Exosome
BPH + PL versus PCa	0.6065	0.6852	0.6758	0.5752	0.6089	0.8189	0.5504	0.5869
BPH versus PL + PCa	0.7175	0.9035	0.7394	0.9062	0.7104	0.9982	0.8260	0.6484

### Exploratory data structure and class clustering (PCA analysis)

**Fig. 5:**
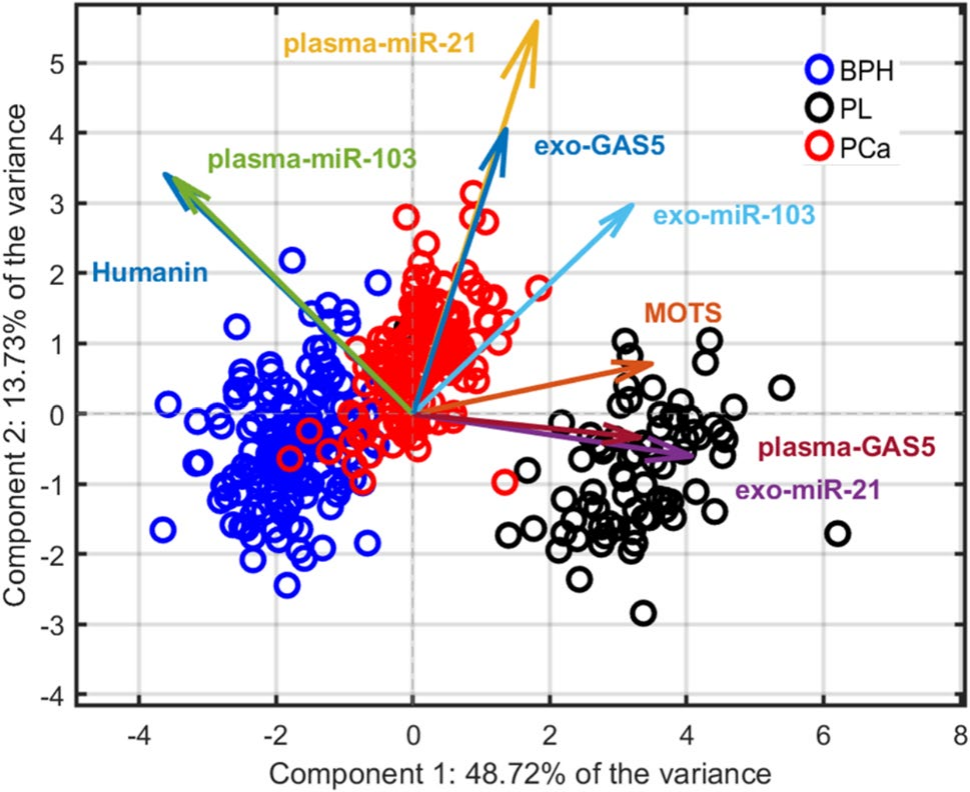
PCA biplot obtained by employing the model on the whole dataset, highlighting the three clinical groups. Loading vectors were scaled tenfold to enhance the visual representation of variable correlations and their associations with class separation

PCA was conducted using all biomarker variables to explore underlying data structure and variable relationships. The PCA biplot (Fig. 5) revealed that the first two principal components (PC1 and PC2) accounted for a cumulative 62.45% of the total variance (PC1: 48.72%; PC2: 13.73%).

The PCA demonstrated clear clustering of the three diagnostic groups, with PC1 driving the separation of PL from both BPH and PCa, and PC2 contributing to the differentiation between BPH and PCa. Notably, Humanin and plasma-miR-103 levels were strongly positively correlated and showed an important negative loading on PC1, indicating that their expression levels contributed most to the variance captured by this component and were key drivers of PL discrimination. In contrast, exo-miR-21, plasma-GAS5 and MOTS are strongly correlated and show a pronounced negative loading on the first principal component, which may reflect an inverse relationship with this component. Conversely, plasma-mir-21 and exo-GAS5 are strongly correlated and primarily aligned along the second principal component, suggesting that these biomarkers may be more informative for distinguish BPH from Pca.

The same analysis has been performed by focusing solely on plasma-derived biomarkers to determine if these more accessible and less technically demanding indicators could also provide meaningful diagnostic insights. This approach aligns with real-world clinical settings, where efficiency and standardization are essential.

**Fig. 6:**
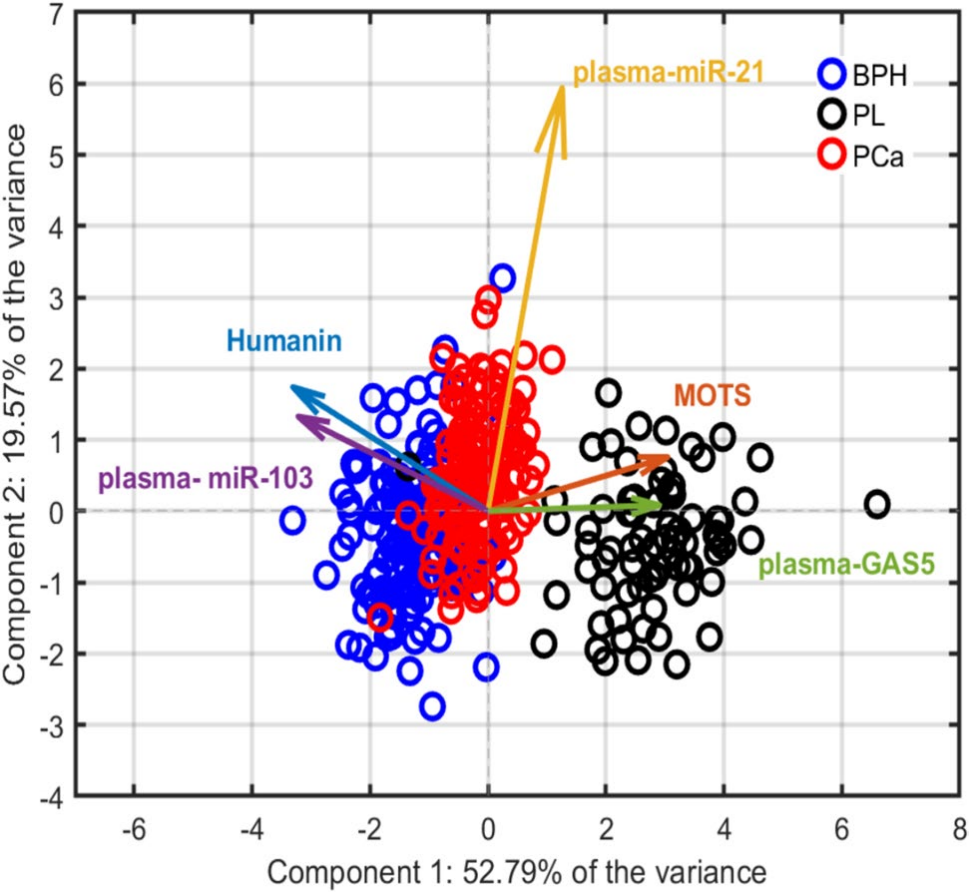
PCA biplot obtained by employing the model on the reduced dataset without the exosomes, highlighting the three clinical groups. Loading vectors were scaled to enhance the visual representation of variable correlations and their associations with class separation

The PCA model obtained with the removal of exosomal variables retained meaningful class separation, as shown in Fig. 6. Although BPH and PCa clusters exhibited increased overlap, PL remained distinctly segregated. The primary contributors to PC1 in this reduced model were Humanin, plasma-miR-103 (negatively correlated), plasma-GAS5, and MOTS (positively correlated). In contrast, plasma-miR-21 made a significant contribution to PC2. These findings highlight distinct molecular profiles across prostate conditions and support the biological relevance of mitochondrial—and miRNA-related markers in class separation, even in the absence of exosomal features.

### Classification modeling and predictive performance

**Fig. 7:**
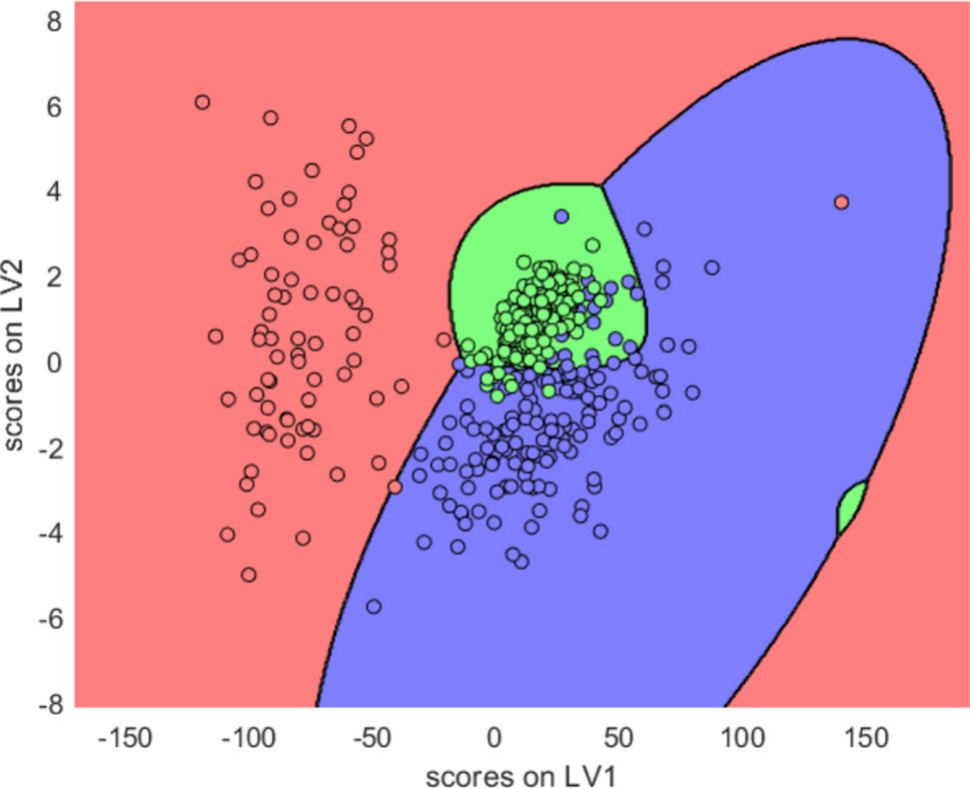
Latent variable score plot derived by PLS, along with the class boundaries determined by quadratic discriminant analysis. Classes 1, 2 and 3 are respectively denoted by blue, red and green circles

**Fig. 8:**
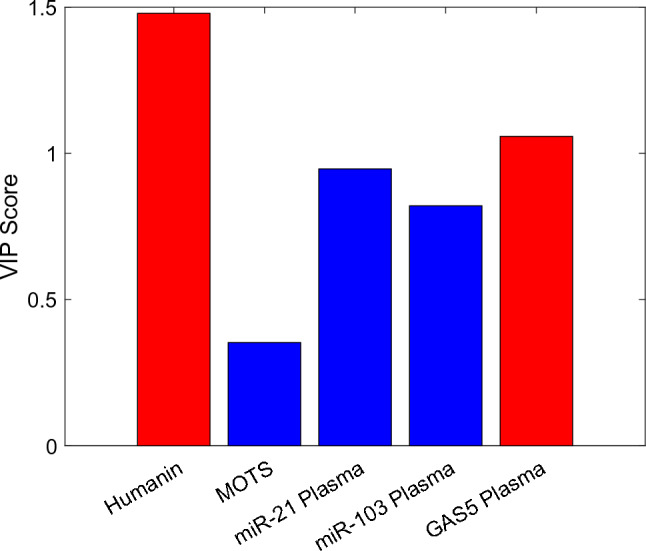
VIP plot for the PLS-DA

To validate the discriminatory potential of circulating biomarkers, supervised machine learning models were applied. PLS-DA was constructed using only plasma-derived variables, specifically Humanin, MOTS-c, GAS5, plasma-miR-21, and plasma-miR-103. The score plot (Fig. 7) shows that the model effectively separated the three clinical groups in latent variable space. Classification boundaries were defined using a quadratic discriminant classifier, achieving a calibration accuracy of 92% and cross-validation accuracy of 91% (1000 Monte Carlo iterations) (Fig. 8).

The Variable Importance in Projection (VIP) analysis (Fig. 5) revealed that plasma levels of Humanin and plasma-GAS5 were the most impactful features in the PLS-DA model. The confusion matrix, Table 6, confirmed high classification accuracy, with correct identification of 28,098 PCa, 14,564 PL, and 24,780 BPH cases, leading to the correct classification in 89.9% of cases.

**Table 6:** PLS-DA-based confusion matrix derived from the classification of the patients belonging to the three clinical groups. The total number of cross validation tests is 75,000

Real/Predicted	BPH	PL	PCa
BPH	24,780	141	5086
PL	376	14,564	32
PCa	1923	0	28,098

The model achieved a sensitivity of 94% in distinguishing PCa from BPH and 89% from PL.

### Classification and regression tree (CART)

**Fig. 9:**
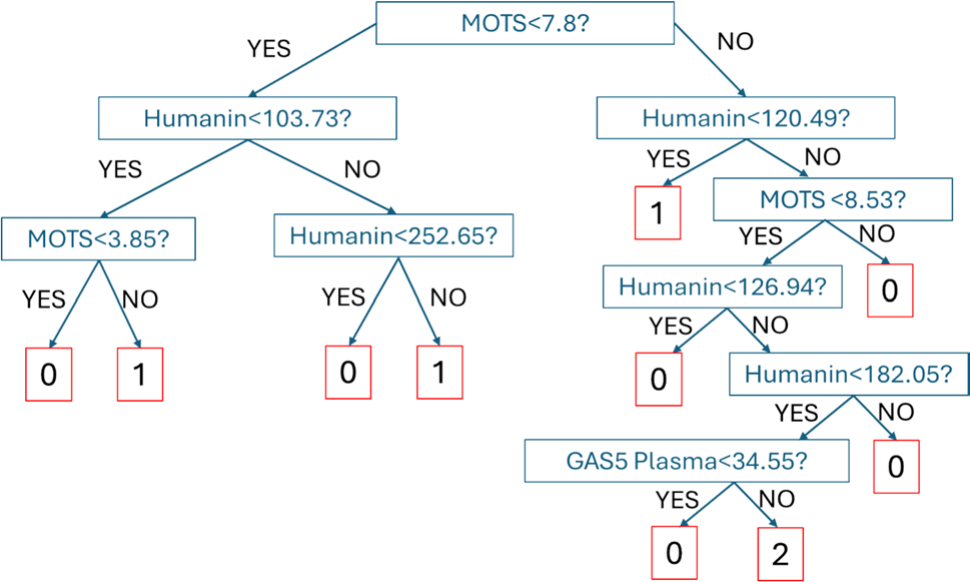
Decision tree model for prostate cancer diagnosis

To improve clinical applicability, a decision tree model was developed (Fig. 6). According to the CART analysis, the final classification relies exclusively on three biomarkers: plasma-GAS5, Humanin, and MOTS. This result partially aligns with the findings from the PLS-DA VIP analysis, which identified Humanin and plasma-GAS5 as the most relevant biomarkers for accurate classification, with VIP scores greater than one. In contrast, MOTS exhibited low VIP values, suggesting a limited role in the multivariate model. Its selection by the CART algorithm, therefore, appears somewhat unexpected. A possible explanation lies in the strong correlation between plasma-GAS5 and MOTS, as illustrated in Fig. 9. In the multivariate PLS-DA model, the predictive contribution of MOTS may be overshadowed by plasma-GAS5 due to this collinearity. Conversely, CART, which is based on univariate decision rules, may treat the two markers independently and thus capture the individual contribution of MOTS more clearly.

This model demonstrated excellent performance, with a calibration accuracy of 99% and cross-validation accuracy of 95%. It accurately identified PL as an intermediate state and effectively differentiated BPH from PCa in the terminal nodes based on GAS5 levels. These results reinforce the potential of non-invasive plasma biomarkers for accurately classifying prostate disease states.

## Discussion

This study advances our understanding of the molecular roles of mitochondrial-derived peptides Humanin and MOTS, the long non-coding RNA GAS5, and the oncogenic microRNAs miR-21 and miR-103 in prostate cancer. The findings support the hypothesis that these circulating biomarkers are differentially regulated across benign prostatic hyperplasia, precancerous lesions, and prostate cancer, highlighting their potential as effective, non-invasive tools for diagnosis and risk stratification [68]. Humanin, a mitochondrial-derived peptide known for its cytoprotective, anti-apoptotic, and anti-inflammatory functions, was found to be significantly downregulated along the progression from BPH to PCa (*p* < 0.0001) [50, 69]. Although Humanin is among the most studied mitokines, its role in cancer biology remains largely unexplored [70, 71]. Mitochondrial function is crucial for tumor cell survival, particularly in managing cellular stress. According to the principle of hormesis, mitochondrial signaling molecules like Humanin may have dual roles: low levels of stress can activate protective pathways, while chronic or intense stress may lead to maladaptive responses [72, 73]. Depending on the intensity and frequency of stress conditions, mitokines can either strengthen the organism (in cases of mild stress) or contribute to metabolic stress in cases of excessive secretion [74].Through an adaptive mechanism, an increase in Humanin concentration attempts to counteract the deleterious effects of mitochondrial degradation [75].

The trend observed in this study aligns with previous observations linking Humanin suppression to increased oxidative stress and mitochondrial dysfunction [50]. Humanin levels are significantly lower in patients with PL and PCa, suggesting a progressive loss of mitochondrial homeostasis during tumor development. Humanin also demonstrated strong discriminative power in distinguishing PL from both BPH and PCa, with statistically significant differences observed (PL vs. BPH, AUC = 0.9855; PL vs. PCa, AUC = 0.9867; both *p* < 0.0001), supporting its potential utility in identifying patients at intermediate risk of malignant progression. Additionally, miR-21 and miR-103, both widely recognized as oncomiRs, were significantly upregulated in PCa, particularly in the exosomal fraction. Exosomal miR-21, a well-known oncogene that promotes cell proliferation and inhibits apoptosis, showed excellent discriminatory performance between BPH and PL (AUC = 0.9625) and between PL and PCa (AUC = 0.9546), both with *p* < 0.0001, supporting its potential role in early tumor development and progression. We are consistent with previous studies highlighting the involvement of miR-21 in PTEN downregulation, apoptosis resistance, and epithelial-mesenchymal transition in PCa. Similarly, miR-103, involved in the regulation of cellular metabolism and tumor progression, was also found to be elevated in the exosomes of PCa patients and has been implicated in metastasis and metabolic reprogramming. The high AUC of exosomal miR-103 across all comparisons confirms its strong diagnostic value and reinforces the emerging concept of exosomal miRNAs as powerful non-invasive biomarkers in oncology.

Particularly noteworthy is the inverse relationship between GAS5 and the oncogenic miRNAs miR-21 and miR-103 (REF). In our study, GAS5 expression—especially in plasma—was significantly reduced in prostate cancer, reinforcing its proposed tumor-suppressor role. This aligns with the "miRNA sponge" mechanism described in previous literature, where GAS5 acts by sequestering oncogenic miRNAs. A decrease in GAS5 may impair this regulatory function, allowing miR-21 and miR-103 to promote tumor progression unchecked. Integrating GAS5 with Humanin and MOTS in multivariate classification models markedly enhanced predictive performance, particularly when coupled with machine learning techniques such as PLS-DA and decision trees.

PCA analysis demonstrate clear segregation of clinical groups, with PL samples occupying a distinct intermediate cluster likely reflecting a transitional molecular phenotype. Importantly, the exclusion of exosomal results did not significantly impair group separation, which has relevant implications for translational applications where plasma-only biomarkers are preferred due to technical feasibility and cost-effectiveness.

In conclusion, the decision tree analysis, based exclusively on three plasma biomarkers (Humanin, MOTS, and GAS5), achieved a cross-validated accuracy of 95%. This finding is clinically relevant, as it enables rapid and interpretable classification through a simplified model, suggesting that the integration of these biomarkers could be valuable for effective patient stratification.

### Limitations and future directions

Despite promising results, this study has several limitations. The lack of longitudinal biomarker data limits insight into their temporal dynamics and external validation in independent and diverse cohorts is needed to confirm the generalizability of our findings. The biomarkers examined are part of a complex regulatory network involving mitochondrial function, cell survival, and prostate cancer progression. Transcriptional and epigenetic regulation studies are needed to clarify the mechanistic interactions between Humanin, GAS5, and oncogenic microRNAs.

## Conclusions

This study highlights the diagnostic potential of Humanin, GAS5 and exosomal miRNAs in distinguishing benign, precancerous and malignant conditions of the prostate. These biomarkers appear to reflect key molecular alterations related to mitochondrial dysfunction, inflammatory signaling, and RNA-mediated regulation. Humanin and GAS5 emerged as candidate tumor suppressors, while exosomal miR-21 and miR-103 showed strong oncogenic profiles and marked discriminatory power. Their integration into multivariate statistical models enabled highly accurate classification, underscoring their translational relevance. Moving forward, longitudinal validation, mechanistic elucidation, and assessment of their utility in treatment monitoring will be essential to advancing non-invasive, biomarker-driven strategies for personalized prostate cancer management.

## Supplementary Information

Below is the link to the electronic supplementary material.

Supplementary file1 (PDF 625 KB)

## Data Availability

The datasets generated and analyzed during this study are available from the corresponding author upon reasonable request. Researchers seeking access may be required to provide a brief proposal outlining the intended use and agree to confidentiality terms as per institutional policies.
