# Supplementary material for: Diagnostic relevance of Humanin, GAS5 and miR-21/miR-103 in prostate disease risk stratification
Source: Clin Exp Med. 2025 Aug 6;25(1):279. doi: 10.1007/s10238-025-01810-z (PMC12328530; doi:10.1007/s10238-025-01810-z)
Supplement: Supplementary file 1 — Supplementary file1 (PDF 625 KB) [file 10238_2025_1810_MOESM1_ESM.pdf]

Diagnostic relevance of Humanin, GAS5 and miR-21/miR-103 in prostate disease risk stratification.

Donatella Coradduzza [1#], Sara Cruciani [1#], Leonardo Sibono [2], Alessandro Tedde [3], Angelo Zinellu [1], Margherita Maioli [1], Alessio Aligi Cogoni [7], Maria Rosaria De Miglio [4], Serenella Medici [5], Massimo Madonia [3], Andrea Angius [6], Massimiliano Grosso [2], Ciriaco Carru [1,7]

1 Department of Biomedical Sciences, University of Sassari, Viale San Pietro 43/B, 07100, Sassari, Italy.
2 Department of Mechanical, Chemical, and Materials Engineering, University of Cagliari, Cagliari, Italy.
3 Department of Medicine, Surgery and Pharmacy, University of Sassari, Sassari, Italy; Unit of Urology, University Hospital of Sassari (A.O.U. SS), Sassari, Italy.
4 Department of Medicine, Surgery and Pharmacy, University of Sassari, Sassari, Italy.
5 Department of Chemical, Physical, Mathematical and Natural Sciences, University of Sassari, Sassari, Italy
6 Institute of Genetic and Biomedical Research (IRGB), National Research Council (CNR), Cittadella Universitaria Cagliari, 09042, Monserrato, CA, Italy.
7 Medical Oncology Unit, University Hospital (AOU) of Sassari, 07100, Sassari, Italy.

# Contributed equally. *

Correspondence: andrea.angius@irgb.cnr.it

Supplementary material

Section S1 - ROC curves for the whole biomarkers set

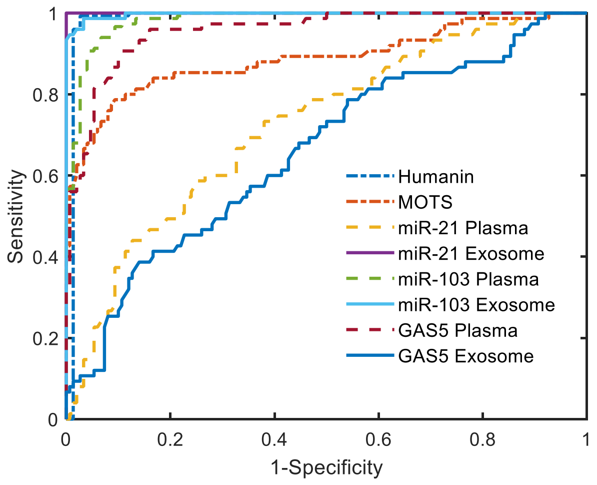

Figure S1.1: ROC curves for the classification task PL vs. BPH. Exosome markers are depicted with solid lines, plasma markers with dashed lines, and mitochondrial peptides Humanin and MOTS with dash-dotted lines.

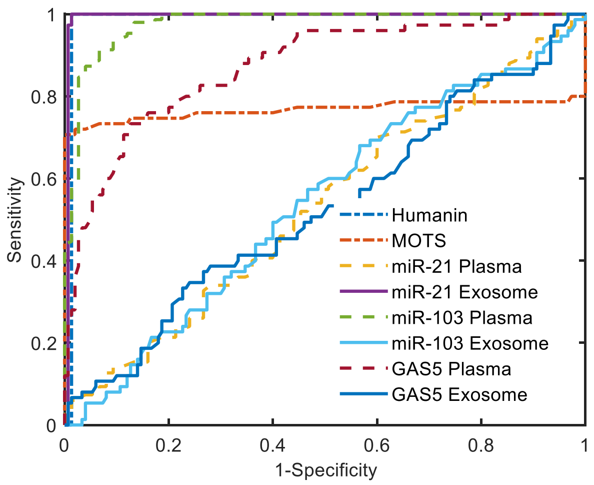

Figure S1.2: ROC curves for the classification task PL vs PCa. Exosome markers are depicted with solid lines, plasma markers with dashed lines, and mitochondrial peptides Humanin and MOTS with dash-dotted lines.

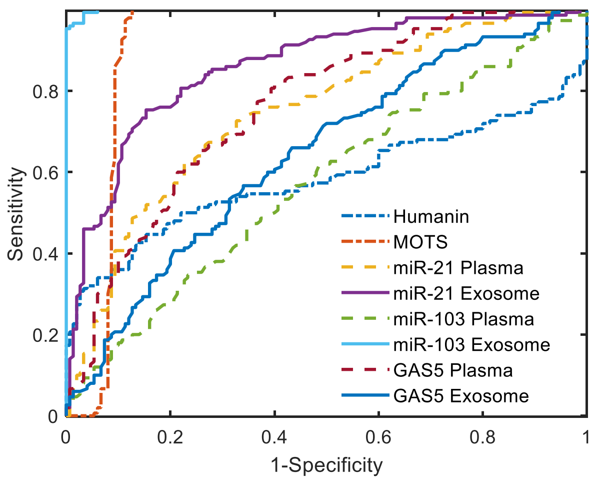

Figure S1.3: ROC curves for the classification task BPH vs PCa. Exosome markers are depicted with solid lines, plasma markers with dashed lines, and mitochondrial peptides Humanin and MOTS with dash-dotted lines.

Section S2 - Delong test

Tables S2.1 and S2.2 present the results of the DeLong test, which was conducted to assess the statistical significance of differences in AUC values across all possible pairs of variables.
Regarding the discrimination between BPH+PL and PCa, the AUC distribution for exosomal miR-103 was significantly different from that of all other variables, as indicated by the low p-values. This finding supports the results reported in the first row of Table 5, highlighting that exosomal miR-103 expression effectively distinguishes PCa from the BPH and PL clinical groups.
In the case of discriminating BPH from PL combined with PCa, Table S2.2 reports smaller p-values. This can be attributed to the high AUC values achieved by MOTS, exosomal miR-21, and exosomal miR-103. MOTS and exosomal miR-21 exhibited statistically equivalent AUCs in distinguishing BPH from the other groups, as indicated by a high p-value (0.91). Despite their good sensitivity, both biomarkers showed significantly lower AUCs compared to exosomal miR-103, as confirmed by the DeLong test p-values ($\sim 10^{-7}$ and $\sim 10^{-10}$, respectively).

Table S2.1: Statistical comparison of AUC values using DeLong's test for each variable, assessing their discriminative performance in the classification task distinguishing BPH + PL from PCa.

Group	Humanin	MOTS	miR-21 Plasma	miR-21 Exosome	miR-103 Plasma	miR-103 Exosome	GAS5 Plasma	GAS5 Exosome
Humanin	-	0.12	0.11	0.55	0.94	1.6e-06	0.26	0.67
MOTS		-	0.80	5.0e-05	0.12	3.0e-07	1.3e-05	0.014
miR-21 Plasma			-	0.0018	0.12	8.8e-06	0.00069	0.025
miR-21 Exosome				-	0.52	1.6e-27	0.36	0.77
miR-103 Plasma					-	1.5e-06	0.22	0.61
miR-103 Exosome						-	9.1e-26	8.3e-12
GAS5 Plasma							-	0.35
GAS5 Exosome								-

Table S2.2: Statistical comparison of AUC values using DeLong's test for each variable, assessing their discriminative performance in the classification task distinguishing BPH to PL + PCa.

Group	Humanin	MOTS	miR-21 Plasma	miR-21 Exosome	miR-103 Plasma	miR-103 Exosome	GAS5 Plasma	GAS5 Exosome
Humanin	-	2.7e-83	1.9e-37	2.5e-80	0.83	3.50e-156	6.4e-50	1.2e-18
MOTS		-	2.4e-07	0.91	1.5e-88	7.5e-07	0.0081	6.7e-13
miR-21 Plasma			-	3.4e-10	2.2e-34	4.0e-23	0.0096	0.019
miR-21 Exosome				-	9.2e-82	8.9e-10	0.0016	3.1e-15
miR-103 Plasma					-	1.8e-162	1.8e-54	1.7e-20
miR-103 Exosome						-	6.4e-16	8.6e-34
GAS5 Plasma							-	6.4e-07
GAS5 Exosome								-

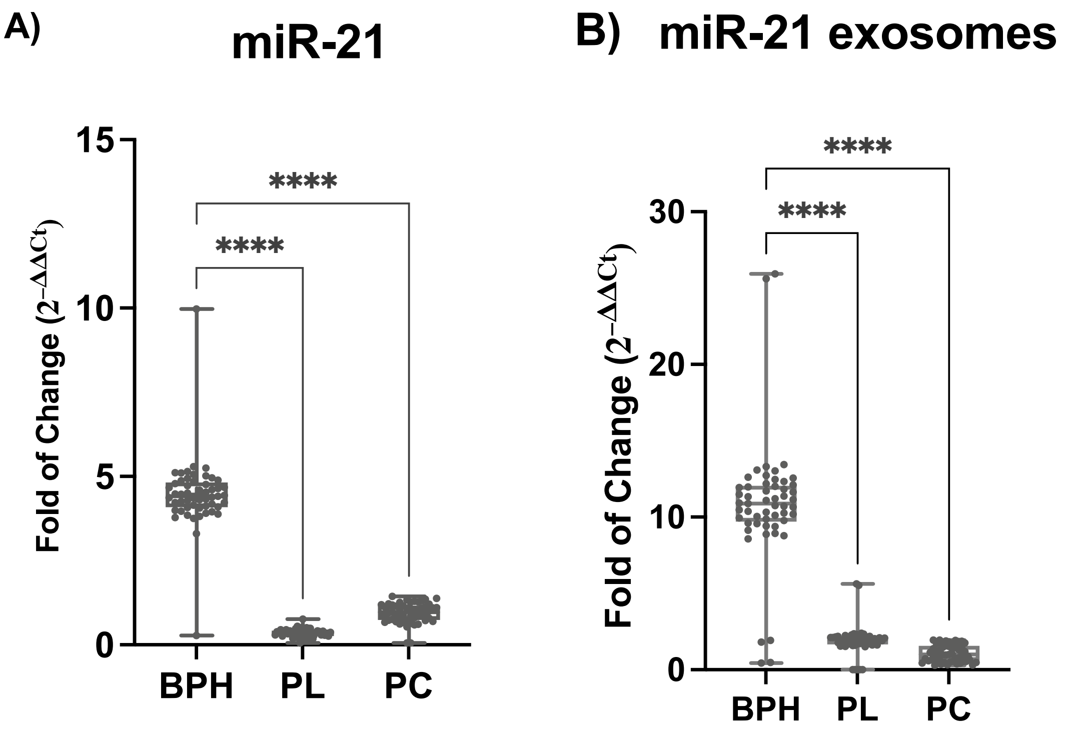

Figure S2.1 and S2.2: Box plots represent expression of miR-21 in plasma and exosomes. The expression of miR-21 in plasma (Panel A) and exosomes (Panel B) were evaluated BPH, PL, and PCa patients. The mRNA levels for each gene were normalized to U6snRNA. The box extends from the 25th to the 75th percentiles, the middle line indicates the median and the whiskers are the 5th and 95th percentiles Data are expressed as mean ± SD, p values refer to Krustal-Wallis test (**** $p \leq 0.0001$).

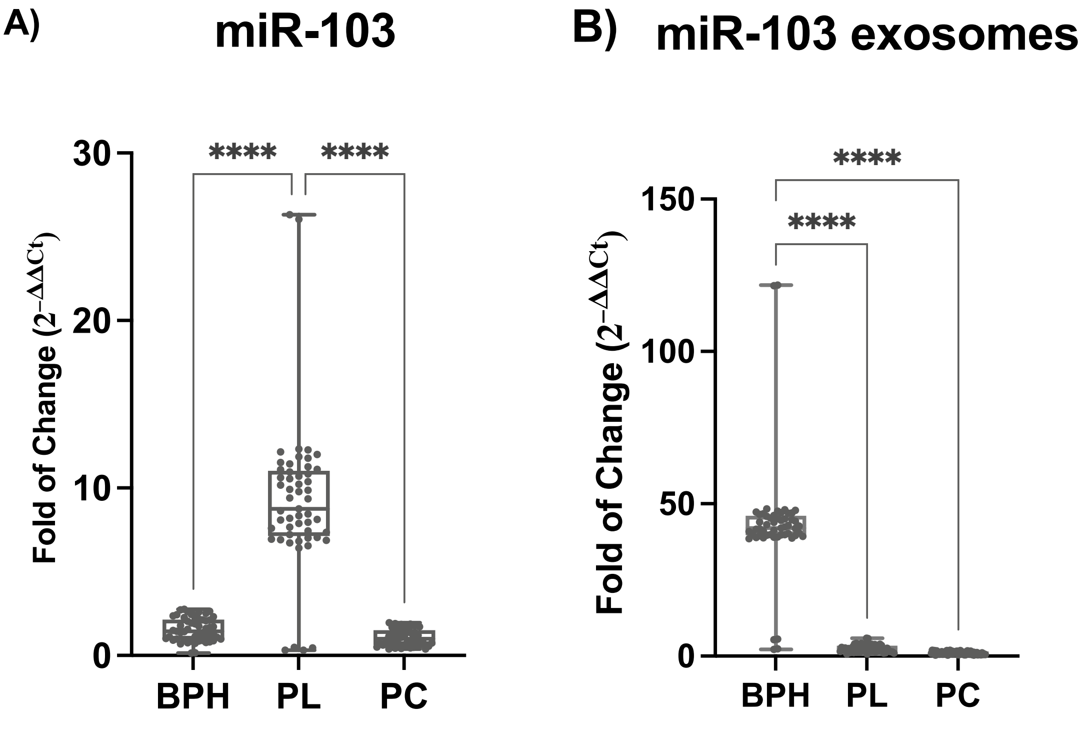

Figure S 2.3 and S2.4: Box plots represent expression of miR-103 in plasma and exosomes. The expression of miR-103 in plasma (Panel A) and exosomes (Panel B) were evaluated BPH, PL, and PCa patients. The mRNA levels for each gene were normalized to U6snRNA. The box extends from the 25th to the 75th percentiles, the middle line indicates the median and the whiskers are the 5th and 95th percentiles Data are expressed as mean ± SD, p values refer to Krustal-Wallis test (* $p \leq 0.05$), (**** $p \leq 0.0001$).

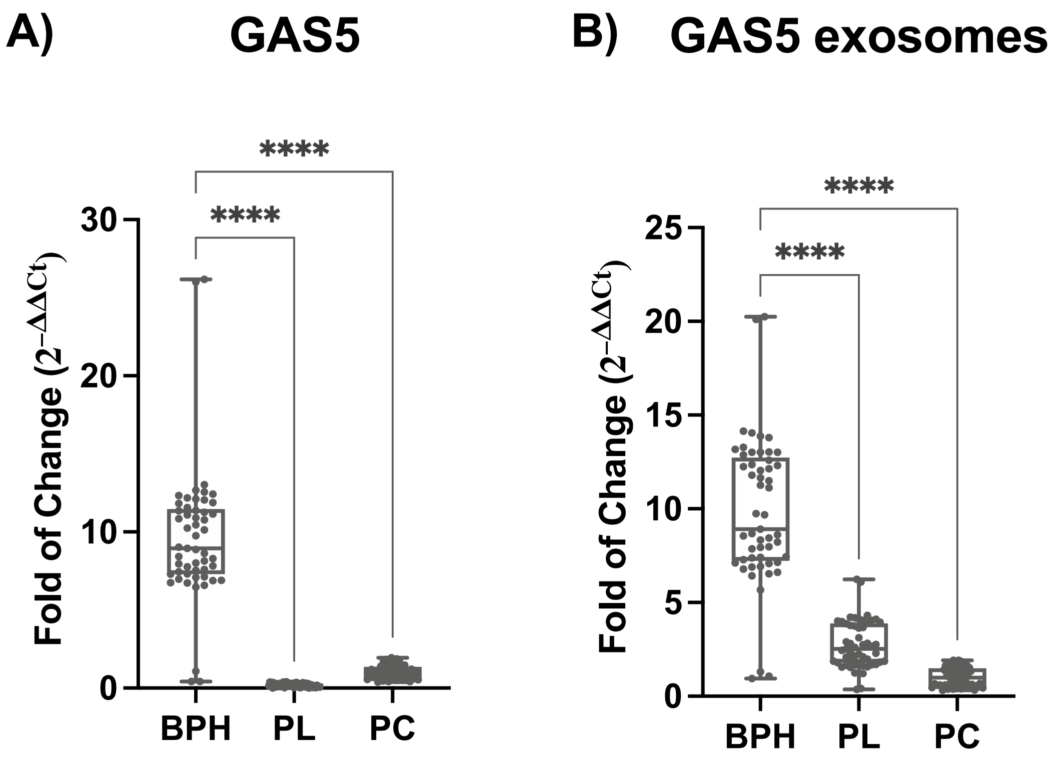

Figure S2.5 and S2.6: Box plots represent expression of Lnc-RNA-GAS5. The expression of GAS5 was evaluated in plasma (Panel A) and exosomes (Panel B) of BPH, PL and PCa patients by qPCR. The mRNA levels for each gene were normalized to Glyceraldehyde-3-Phosphate-Dehydrogenase (GAPDH). The box extends from the 25th to the 75th percentiles, the middle line indicates the median and the whiskers are the 5th and 95th percentiles Data are expressed as mean ± SD, p values refer to Krustal-Wallis test (** $p \leq 0.01$), (**** $p \leq 0.0001$).
